# Supplementary figures and images for: Programming effects of peripubertal stress on spatial learning
Source: Neurobiol Stress. 2020 Dec 1;13:100282. doi: 10.1016/j.ynstr.2020.100282 (PMC7739188; doi:10.1016/j.ynstr.2020.100282)

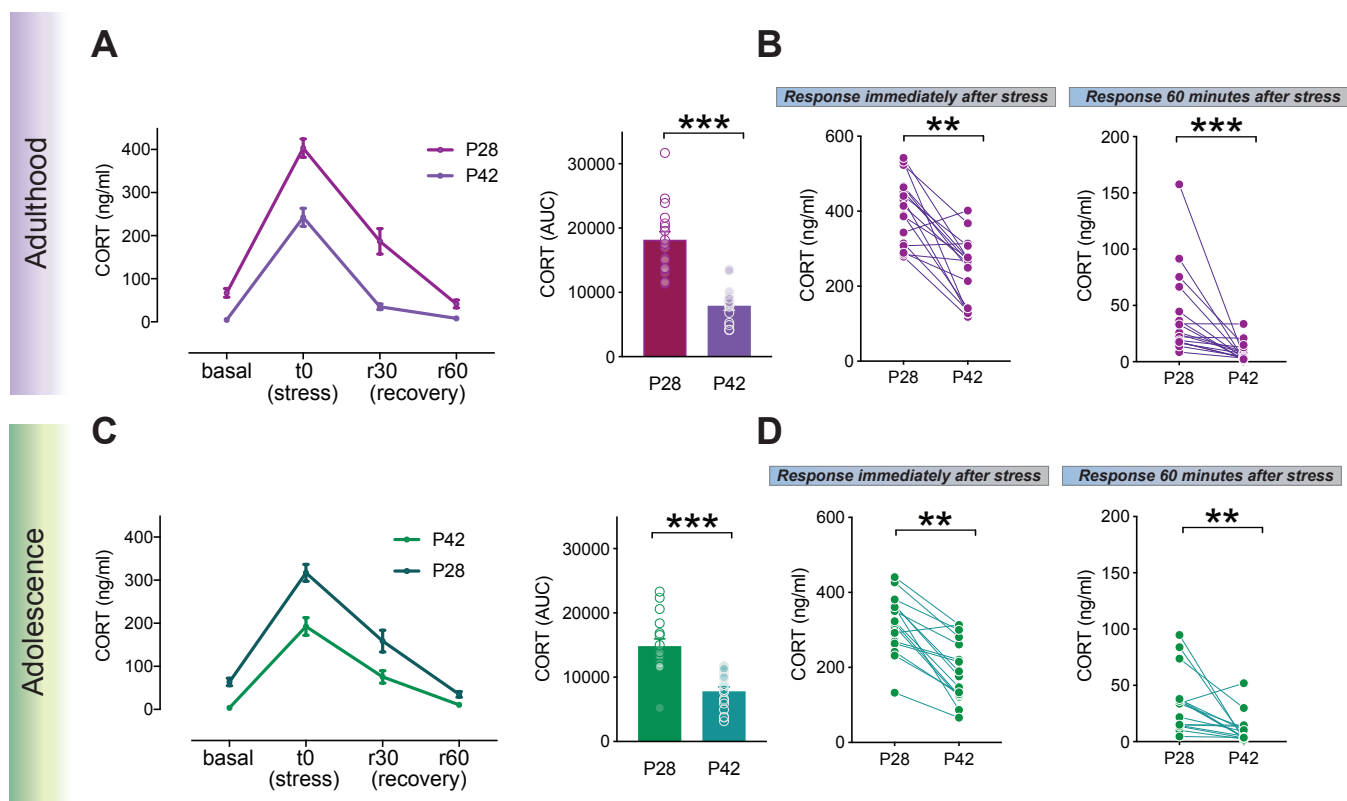

Figure S1

Supplement: Multimedia component 1 [file mmc1.pdf]
